# Dysglycemia, gender, and cognitive performance in older persons living with mild cognitive impairment: findings from a cross-sectional, population-based study

**DOI:** 10.1007/s40520-024-02806-7

**Published:** 2024-07-16

**Authors:** Virginia Boccardi, Emma Giulia Travaglini, Emanuela Sciacca, Francesca Mancinetti, Ilenia Murasecco, Anna Giulia Guazzarini, Patrizia Bastiani, Carmelinda Ruggiero, Patrizia Mecocci

**Affiliations:** 1 Division of Gerontology and Geriatrics, Department of Medicine and Surgery, University of Perugia, Piazzale Gambuli 1, Perugia, 06132 Italy; 2 Division of Clinical Geriatrics, Department of Neurobiology, Care Sciences and Society, Karolinska Institutet, Stockholm, Sweden

**Keywords:** Aging, Cognition, Dysglycemia, Gender, Prevention

## Abstract

**Objective:**

This study aims to examine the relationship between dysglycemia - also known as pre-diabetes or impaired glucose tolerance- and cognitive abilities in an older population living Mild Cognitive Impairment (MCI) and stratified by gender.

**Study design:**

This is a retrospective study with data gathered from a large Italian clinical-based database.

**Main outcome measures:**

The evaluation of cognitive performances by the Mini-Mental State Examination and the Addenbrooke’s Cognitive Examination Revised rating scale as tests of screening and a comprehensive neuropsychological evaluation of several cognitive areas.

**Results:**

The study comprised 682 subjects (445 F/237 M) with a mean age of 76.08 ± 9.03 (range: 66–93) years. In all population, subjects with dysglycemia 193 (28.3%) had significantly poorer performance in memory (*p* = 0.006) and logic reasoning (*p* = 0.007) when compared with subjects without dysglycemia. The linear regression analyses revealed significant differences in the correlates of cognitive domains between gender groups. Independent of multiple covariates, women with dysglycemia showed worse performances in attention and short-term memory domains as compared with men. Even in the absence of dysglycemia women were more likely to show lower score in screening test of general cognition and attention.

**Conclusions:**

Our findings suggest that dysglycemia in older individuals with MCI is associated with declines in specific cognitive domains, potentially influenced by gender. Implementing a comprehensive approach involving risk stratification and preventive strategies may be more effective in averting further cognitive decline in this high-risk population.

**Supplementary Information:**

The online version contains supplementary material available at 10.1007/s40520-024-02806-7.

## Introduction

Clinical and epidemiological studies have suggested a potential association between type 2 diabetes mellitus (T2DM) and cognitive decline along with aging [1, 2]. Based on current evidence, dementia is conceptualized as a biological and clinical continuum from preclinical to clinical or symptomatic phases. Mild Cognitive Impairment (MCI) delineates the early stage of cognitive decline preceding dementia and is characterized by a complex interplay of symptom intensity and neuropsychological functioning [3]. Dementia progression varies widely among individuals, and the factors predicting cognitive and functional decline in cases of rapid progression are not yet well-defined. In this context, much evidence shows that hyperglycemia and uncontrolled T2DM are well-known risk factors for the acceleration of cognitive decline, MCI, and the development of dementia [4].

Dysglycemia - also known as pre-diabetes or impaired glucose tolerance- is a medical condition where an individual’s blood glucose levels are higher than normal but not yet high enough to be classified as diabetes [5, 6]. The presence of dysglycemia serves as a warning sign, indicating an increased risk of developing T2DM in the future. Despite compelling epidemiological data indicating an elevated dementia risk in individuals with diabetes [7], the relationship between dysglycemia and cognitive impairment remains a topic of debate, with some studies indicating an association while others do not find a clear link [7, 8]. However, recent studies suggest that dysglycemia may be linked to poorer cognitive performance, particularly in domains such as processing speed [9], while others have not substantiated these findings [10]. A cross-sectional population-based study showed that having pre-diabetes or diabetes is associated with deficits in global cognitive function, processing speed, and executive functioning compared to having normal glucose tolerance [11]. Another 12-year longitudinal study showed that long-term dysglycemia is a risk factor for a faster cognitive decline during aging, impacting verbal fluency performances [12].

Interestingly, variations in cognitive function between genders have also been suggested, even if findings have been limited and inconsistent. Prior meta-analysis exploring gender disparities in the connection between T2DM, and dementia revealed a notably tighter association in women compared to men [13]. Moreover, a study by Verhagen and colleagues demonstrated that women with T2DM faced a heightened risk of accelerated cognitive decline in a 6-year follow-up, surpassing the risk observed in men [14]. Another population-based cross-sectional cohort study (age range 40–75 years old) has shown that T2DM, but not dysglycemia, is associated with higher odds of cognitive impairment [15, 16]. The connection between dysglycemia and cognitive impairment in old populations and the prodromal phase of dementia, along with any gender differences, remains largely unknown. Early detection of cognitive changes through neuropsychological screening and tests is essential in this context for implementing future interventions aimed at mitigating adverse outcomes and enhancing problem management. This study aims to evaluate whether there is a gender-specific association between dysglycemia and cognitive performance in a large cohort of older individuals living with MCI.

## Methods

This is a retrospective study with data gathered from the GeriCo study Geriatric Cognitive Evaluation login (https://gericoev.eu), a large Italian clinical-based study at the Geriatric section of the University of Perugia focused on cognitive impairment and dementia in old age subjects. From a total of 1850, only subjects with a diagnosis of MCI and without a diagnosis of any diabetes have been considered. Criteria for MCI [17] thus were broadened to consider both non-amnestic presentations and the involvement of multiple cognitive domains but continued to require “essentially normal” functional activities. Criteria for MCI include [17]: (1) change in cognition recognized by the affected individual or observers; (2) objective impairment in one or more cognitive domains; (3) independence in functional activities; and (4) absence of dementia. All participants recruited provided informed consent, and the study adhered to the Declaration of Helsinki and was approved by the Regional Ethical Committee (Prot. n. 8005/16/ON).

### Cognitive performances assessment

Trained psychologists assessed participants in a quiet, comfortable room through an ad hoc neuropsychological battery. The Mini-Mental State Examination, the Addenbrooke’s Cognitive Examination-Revised (ACE-R) and the Clock Drawing Test were used as screening tests [18]. Afterward, participants were evaluated using a detailed neuropsychological battery (see Supplemental Table 1) to assess different cognitive functions in more detail. *Attention* was measured using a cancellation task (Attentional Matrices) and Trail Making Test A (TMT A). Different measures were selected to catch various aspects of *memory*: (1) the Digit Span Forwards test was selected for the ability to maintain visuospatial information for a brief time; (2) the Digit Span Backward test as a measure of working memory’s central executive; (3) the Rey Auditory Verbal Learning Test as a measure of verbal learning and memory; (4) the Prose Memory test by Babcock story recall as a measure of memory for structured verbal information (5) the Corsi Span tasks to assess verbal and visuospatial short-term memory. Regarding *language ability*, the letter (FAS) and Categories Fluency tests were inserted to measure verbal fluency involving both linguistic skills and executive functions. The Token test was used to measure the comprehension level. *Executive functions* were measured using the Trail Making Test B (TMT B). Raven’s test was used as a measure of *fluid intelligence and logical reasoning.* Details on administration procedures, Italian normative data for score adjustment for age and education, and normality cut-off scores are available.

Hachinski Ischemic Score (HIS) was also administered. This scoring system was composed of thirteen items: acute onset, stepwise deterioration, fluctuating symptoms, nocturnal confusion, relative preservation of personality, depression, somatic disturbances (non-focal neurological signs and symptoms), emotional lability (spastic laughter and crying), hypertension, previous cerebrovascular accident (stroke), lateralized focal symptoms, lateralized focal signs, signs of atherosclerosis in other areas. HIS was originally developed to establish a relationship between cerebral blood flow and dementia. Researchers have recognized the utility of the HIS as a tool to differentiate ischemic forms of dementia from AD cases. The HIS also possesses good psychometric properties and is related to cognitive functioning in MCI [19, 20]. The HIS is also associated with various vascular factors and cognitive scales in community-dwelling older adults. It appears to aid in evaluating the extent of vascular factors and predicting cognitive function status [20].

### Clinical and biochemical variable assessment

Prediabetic (dysglycemic) status was ascribed based on blood draw from the baseline visit and was defined as fasting glucose blood levels of 100–125 mg/dL based on guidelines from the American Diabetes Association [21]. Anthropometric determinations (weight, height, and BMI) were measured using standard techniques. BMI was calculated as weight in kilograms divided by the square of height expressed in meters (Kg/m^2^). Systolic and diastolic blood pressure was measured twice on the right upper arm in the sitting position using a manual sphygmomanometer, and the mean value was used for the analyses. Blood samples were collected in the morning after fasting overnight. Blood glucose, total cholesterol, and triglycerides were analyzed using enzymatic methods, whereas high-density lipoprotein (HDL)-cholesterol was measured after isolation of low-density lipoprotein (LDL) and very-low-density lipoprotein (VLDL; Boehringer Mannheim GmbH. Germany) and LDL-cholesterol was calculated using Friedewald’s method. Vitamin B12, albumin, calcium and folic acid were measured by enzymatic method.

### Statistical analysis

The observed data were normally distributed and are presented as mean± Standard Deviation (SD) or Standard Error (SE), where appropriate. Descriptive statistics were used to describe participant characteristics related to the variables studied. The χ^2^ test and the independent t-test were used to compare the differences in characteristics between the two groups. Analysis of covariance (ANCOVA) by general linear model was used to compare differences in cognitive function scores between the two groups, with age and years of education as covariates. The independent t-test was used to assess differences between variables in two separate groups (dysglycemia/no dysglycemia; men/women). A series of multiple regression analyses performed by General Linear Models were used to detect the factors correlated with performance in different cognitive domains again for the two groups of dysglycemia/no dysglycemia. Age, years of education, systolic blood pressure, HIS, total cholesterol, LDL-cholesterol, HDL-cholesterol, triglycerides and antithrombotic drugs were included in the models as covariates. According to a global effect size of 25% with type I error of 0.05 has a power of 98% (GPower 3.1.7). All *p* values are two-tailed, and the level of significance was set at *p* ≤ 0.05. Statistical analyses were performed using the SPSS 21 software package (SPSS. Inc. Chicago. IL).

## Results

### Sample characteristics

The sample population includes 682 subjects, mostly women (445; 65.2%) with a mean age of 76.08 ± 9.03 (range: 66–93) years. Table 1 shows the clinical characteristics of the sample population stratified by gender. Women had significantly lower years of education (9.58 ± 5.07 vs. 10.51 ± 5.04, *p* = 0.022), higher total cholesterol (215.51 ± 39.26 vs. 191.55 ± 37.95, *p* < 0.0001), higher HDL-cholesterol levels (62.90 ± 14.77 vs. 52.78 ± 13.92. *p* < 0.0001), higher LDL-cholesterol (127.86 ± 33.45 vs. 117.02 ± 34.19, *p* = 0.001) as well as lower HIS (1.88 ± 1.48 vs. 2.23 ± 1.90, *p* = 0.016) as compared with men. No other statistically significant differences were found in the examined variables. As far as clinical story is concerned, no difference was found in the story of hypertension, stroke, myocardial infarction, or dyslipidemia (data not shown). 104 (23.3%) women and 51 (21.5%) men used anti-lipid drugs (*p* = 0.687); 260 (58.4%) woman and 142 (59.9%) men (*p* = 0.443) used anti-hypertensive drugs with no gender difference. Instead, women (115; 25.8%) as compared with men (81; 34.1%) were significantly less likely to use anti-thrombotic drugs (*p* = 0.013).

**Table 1:** Characteristics of the total sample population, stratified by gender (*n* = 682)

	Total (*n* = 682)	Women (*n* = 445)	Men (*n* = 237)	*p*
Age (years)	76.08 ± 9.03	75.75 ± 9.59	76.70 ± 7.83	0.192
Education (years)	9.90 ± 5.08	9.58 ± 5.07	10.51 ± 5.04	**0.022**
DBP (mmHg)	74.02 ± 10.29	74.25 ± 10.44	73.56 ± 10.02	0.450
SBP (mmHg)	131.33 ± 18.15	131.23 ± 17.12	131.52 ± 20.02	0.856
Cholesterol total (mg/dl)	206.83 ± 40.43	215.51 ± 39.26	191.55 ± 37.95	**< 0.0001**
HDL-C (mg/dl)	59.21 ± 15.25	62.90 ± 14.77	52.78 ± 13.92	**< 0.0001**
LDL-C (mg/dl)	123.80 ± 34.09	127.86 ± 33.45	117.02 ± 34.19	**0.001**
Triglycerides (mg/dl)	113.60 ± 48.57	115.70 ± 48.02	109.93 ± 49.42	0.178
Glucose (mg/dl)	99.53 ± 16.76	98.57 ± 16.34	101.23 ± 17.39	0.073
Vitamin B12 (pg/ml)	285.00 ± 190.32	287.31 ± 166.72	281.08 ± 225.29	0.728
Albumin (g/dl)	4.05 ± 0.22	4.05 ± 0.21	4.06 ± 0.24	0.882
Calcium (mg/dl)	9.54 ± 0.55	9.67 ± 0.56	9.33 ± 0.48	0.067
Folic acid (ng/ml)	10.72 ± 6.94	11.49 ± 6.94	9.06 ± 692	0.236
HIS	2.00 ± 1.65	1.88 ± 1.48	2.23 ± 1.90	**0.016**

DBP: diastolic blood pressure; SBP: systolic blood pressure; HDL-C: high-density lipoprotein cholesterol; LDL-C: low-density lipoprotein cholesterol. HIS: Hachinski Ischemic Score

28.2% (*n* = 193) of the total sample population had dysglycemia with no difference between gender. Table 2 shows the clinical characteristics of the sample population stratified by the presence of dysglycemia. Subjects with dysglycemia were older (78.29 ± 7.03 vs. 75.20 ± 9.57, *p* < 0.0001), had higher systolic blood pressure (134.09 ± 17.50 vs. 130.26 ± 18.30, *p* = 0.024), lower HDL-cholesterol levels (57.35 ± 13.51 vs. 60.81 ± 16.02, *p* = 0.039) as well as higher triglycerides (121.24 ± 52.85 vs. 109.67 ± 45.79, *p* = 0.008) as compared with subjects without dysglycemia. No statistically significant differences were found in all the other variables examined. No significant difference was found in the history of hypertension, stroke, myocardial infarction, or dyslipidemia between groups, as well as in the use of anti-lipid, anti-hypertensive, and anti-thrombotic drugs (data not shown).

**Table 2:** Characteristics of the total sample population stratified by glycemic values (*n* = 682)

Dysglycemia	(*n* = 193)	No dysglycemia (*n* = 489)	*p*
Gender (M/F)	72/121	165/324	0.214^*^
Age (years)	78.29 ± 7.03	75.20 ± 9.57	**< 0.0001**
Education (years)	9.40 ± 4.92	10.10 ± 5.13	0.105
DBP (mmHg)	74.97 ± 10.27	73.65 ± 10.29	0.171
SBP (mmHg)	134.09 ± 17.50	130.26 ± 18.30	**0.024**
Cholesterol total (mg/dl)	210.51 ± 42.27	204.91 ± 39.36	0.120
HDL-C (mg/dl)	57.35 ± 13.51	60.81 ± 16.02	**0.039**
LDL-C (mg/dl)	127.32 ± 34.55	122.12 ± 33.80	0.139
Triglycerides (mg/dl)	121.24 ± 52.85	109.67 ± 45.79	**0.008**
Glucose (mg/dl)	108.10 ± 7.16	94.95 ± 18.54	**< 0.0001**
Vitamin B12 (pg/ml)	276.51 ± 196.39	289.65 ± 187.06	0.467
Albumin (g/dl)	4.00 ± 0.10	4.09 ± 0.29	0.226
Calcium (mg/dl)	9.64 ± 0.49	9.48 ± 0.58	0.386
Folic acid (ng/ml)	11.53 ± 7.64	10.29 ± 6.60	0.536
HIS	2.21 ± 1.35	1.93 ± 1.74	0.073

DBP: diastolic blood pressure; SBP: systolic blood pressure; HDL-C: high-density lipoprotein cholesterol; LDL-C: low-density lipoprotein cholesterol. HIS: Hachinski Ischemic Score. *χ^2^ = 0.775

The clinical characteristics of the sample population, separated into women and men stratified by glycemic status, are reported in Supplemental Tables 2 and 3. In women, subjects with dysglycemia were older (78.50 ± 7.38 vs. 74.72 ± 10.12, *p* < 0.0001) with significantly lower years of education (8.69 ± 4.73 vs. 9.91 ± 5.16, *p* = 0.025), higher diastolic blood pressure (76.13 ± 10.52 vs. 73.54 ± 10.34, *p* = 0.033) and systolic blood pressure (135.02 ± 17.54 vs. 129.79 ± 16.76, *p* = 0.008) as well as higher total cholesterol (223.15 ± 40.83 vs. 211.60 ± 37.92, *p* = 0.008) and triglycerides (127.39 ± 54.84 vs. 109.78 ± 43.11, *p* = 0.001). No statistically significant differences were found in men for any of the other variables examined and reported.

### Differences in cognitive performances by groups of dysglycemia and gender

**Fig. 1:**
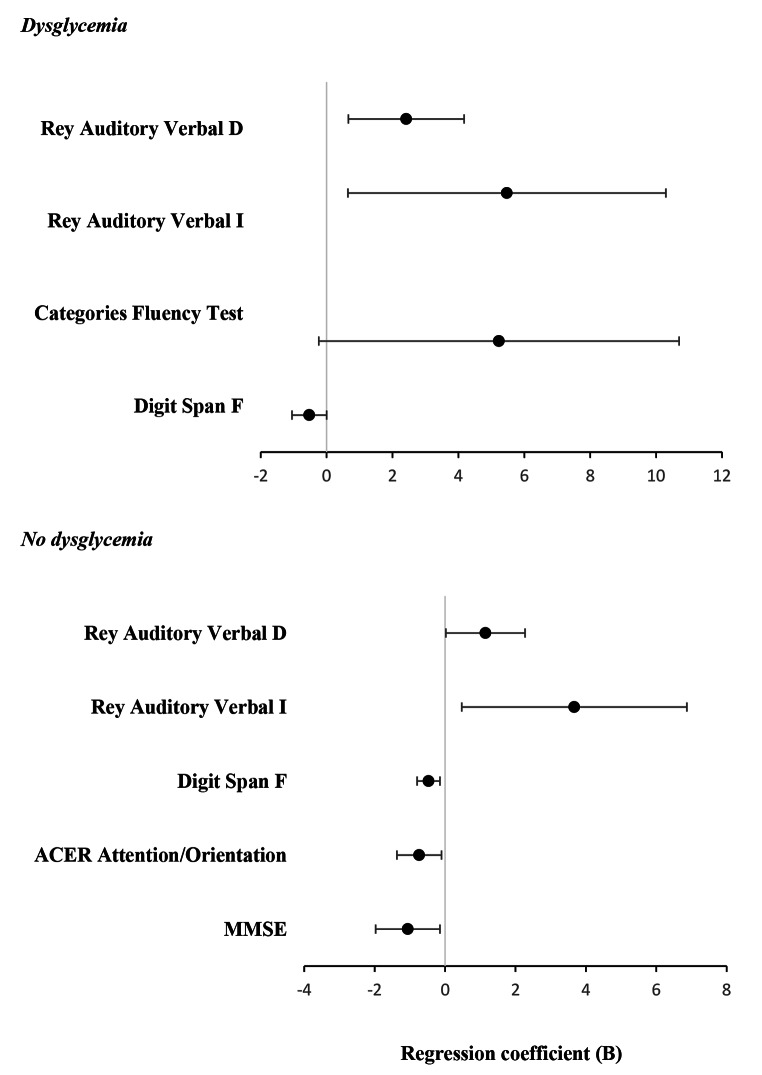
Association between gender and cognitive performances among subjects living with MCI in the groups of dysglicemia Associations between gender and cognitive performances. B coefficients were estimated using linear regression models. Models are adjusted for age, years of education, systolic blood pressure, Hachinski Ischemic Score, total cholesterol, high-density lipoprotein cholesterol, low-density lipoprotein cholesterol, triglycerides, and antithrombotic drugs use. Gender is indicated as M = 1, F = 2; antithrombotic drugs use indicated as yes or no (1 or 0) Rey Auditory Verbal D: Delayed recall and I: Immediate recall; ACE-R: Addenbrooke’s Cognitive Examination-Revised; Digit Span F: forward; MMSE, Mini-Mental State Examination

In all population, subjects with dysglycemia had significantly poorer performances in the Babcock story recall (4.45 ± 0.49 vs. 6.05 ± 0.29, *p* = 0.006) and Raven’s test (23.64 ± 0.43 vs. 25.06 ± 0.27, *p* = 0.007) as compared with subjects without dysglycemia. A trend was found in the Letter Fluency test (Table 3). Linear regression analyses revealed significant differences in the correlates of cognitive domains between groups after correction for multiple confounding variables (Fig. 1). Among all domains explored, models controlled by age, years of education, systolic blood pressure, HIS, total cholesterol, LDL-cholesterol, HDL-cholesterol, triglycerides and anti-thrombotic drugs revealed that in the presence of dysglycemia being woman was associated only with lower scores in Digit Span Forward (β=-0.250, *p* = 0.05; R^2^ = 0.125) as well as higher score in Rey Auditory Verbal Immediate (β = 0.261, *p* = 0.027; R^2^ = 0.320) and Rey Auditory Verbal Delayed (β = 0.330, *p* = 0.008; R^2^ = 0.262). A trend was found for the Fluency Categories test (β = 0.493, *p* = 0.051; R^2^ = 0.375).

**Table 3:** Neuropsychological assessment of the total sample population, stratified by glycemic values (*n* = 682)

	Dysglycemia (*n* = 193)	No dysglycemia (*n* = 489)	*p*
***Screening***			
MMSE	26.42 ± 0.22	26.61 ± 0.13	0.481
ACE-R	76.17 ± 0.79	76.21 ± 0.50	0.970
ACE-R Attention/Orientation	16.48 ± 0.13	16.22 ± 0.08	0.115
ACE-R Memory	5.08 ± 0.22	5.29 ± 0.14	0.422
ACE-R Language	22.54 ± 0.25	22.56 ± 0.16	0.941
ACE-R Fluency	7.79 ± 0.18	8.09 ± 0.11	0.174
ACE-R Visuo-spatial	13.13 ± 0.17	13.26 ± 0.10	0.537
Clock Drawing Test	3.61 ± 0.10	3.64 ± 0.06	0.863
***Attention***			
Attentional Matrices	40.93 ± 0.73	41.71 ± 0.45	0.375
Trail Making Test A	71.86 ± 4.29	78.27 ± 2.68	0.209
***Memory***			
Digit Span Forward	4.94 ± 0.07	5.10 ± 0.04	0.076
Digit Span Backward	3.22 ± 0.91	3.50 ± 1.01	0.102
Rey Auditory Verbal I	29.94 ± 0.75	30.63 ± 0.47	0.442
Rey Auditory Verbal D	5.04 ± 0.26	5.06 ± 0.16	0.950
Babcock story recall	4.45 ± 0.49	6.05 ± 0.29	**0.006**
Corsi Span	4.17 ± 0.07	4.29 ± 0.04	0.160
***Language***			
Letter Fluency Test (FAS)	27.07 ± 0.81	28.81 ± 0.51	0.075
Categories Fluency Test	14.39 ± 0.86	15.96 ± 0.51	0.127
Token test	31.06 ± 0.30	31.19 ± 0.20	0.723
***Executive Functions***			
Trail Making Test B	194.03 ± 12.72	202.43 ± 7.89	0.579
***Fluid Intelligence and Logic Reasonings***			
Raven’s test	23.64 ± 0.43	25.06 ± 0.27	**0.007**

MMSE: Mini-Mental State Examination; ACE-R: Addenbrooke’s Cognitive Examination. Rey Auditory Verbal I: Immediate recall, D: Delayed recall. The scores are corrected by age and years of education by ANCOVA. Data are presented as means ± standard errors

In subjects without dysglycemia, among all neuropsychological tests examined independent of the multiple covariates indicated, being women was associated with lower scores in MMSE (β=-0.163, *p* = 0.023; R^2^ = 0.206), ACER Attention/Orientation (β=-0.171, *p* = 0.023; R^2^ = 0.153) and Digit Span Forward (β=-0.218, *p* = 0.05; R^2^ = 0.120) as well as higher score in Rey Auditory Verbal Immediate (β = 0.166, *p* = 0.025; R^2^ = 0.271) and Rey Auditory Verbal Delayed (β = 0.154, *p* = 0.046; R^2^ = 0.204) tests.

## Discussion

Dysglycemia, also referred to as pre-diabetes, has been increasing globally along with aging. The number of people affected worldwide is estimated to reach 472 million by the year 2025 [22]. People with dysglycemia have an increased risk of developing non only T2DM, but also other conditions, including the risk of cognitive decline and related dementia [23]. Cognitive function encompasses a spectrum of mental faculties facilitating interaction and navigation within one’s environment, such as perception, memory encoding and retrieval, problem-solving, reasoning, and communication. Despite being conventionally perceived as a cohesive entity, cognitive function can be parsed into different domains, each representing a distinct facet of mental processing. Understanding the complex influence of dysglycemia on these cognitive domains is important for a comprehensive understanding of its impact on individuals’ lives and for the development of tailored interventions aimed at preventing cognitive decline. Our study including 682 older subjects (mostly women) living with MCI, collectively shows that 1 person every 3.5 has dysglycemia with no gender difference. Overall, subjects with dysglycemia show poorer performances in memory (evaluated by the Babcock story recall), logic reasoning, and fluid intelligence (evaluated by the Raven’s test) compared to subjects with normal glycemic values. MCI is a condition in which people experience more memory or thinking problems than others their age, and it is considered an intermediate stage in the trajectory from normal cognition to dementia [24]. However, MCI is a heterogeneous disorder with a different prognosis for progression to dementia. Previous studies have shown that the risk of conversion to dementia is higher in subjects with impairment in the memory domain compared to other subtypes [24, 25]. The Babcock story recall test is a neuropsychological assessment tool used to evaluate verbal memory, particularly the ability to recall narrative information. Thus, the fact that subjects with dysglycemia have poorer performance in the memory domain may be related to a higher risk of conversion, and further studies are necessary to confirm such a hypothesis. Raven’s Test, instead, is a non-verbal test typically used to measure global intelligence and abstract reasoning and is regarded as a non-verbal estimate of fluid intelligence. This novel finding can be related to alteration in specific area of brain more susceptible to glucose levels variation. Fluid intelligence is associated with several brain characteristics and markers of brain health [26]. Research indicates that individuals with damage to the prefrontal and parietal cortex tend to perform worse on fluid intelligence assessments compared to those without such damage [27]. Additionally, heightened activity in the frontal, parietal, and anterior cingulate cortices has been observed during tasks requiring fluid reasoning. Accordingly, in a small study involving 23 participants with prediabetes, researchers found that higher levels of insulin resistance were associated with decreased cerebral metabolic rate of glucose in the frontal, parietotemporal, and cingulate brain regions [28]. Again, a recent meta-analysis revealed that prediabetes is inversely associated with grey matter volume and white matter volume [29] It is possible to hypothesize that dysglicemic status can lead to endothelial dysfunction within the cerebral microcirculatory system, contributing to deficits in cerebral perfusion and the onset of chronic hypometabolism [30]. According to this hypothesis a recent longitudinal study of non-demented older adults has shown that pre-diabetes is associated with brain hypometabolism and cognitive decline in a gender-dependent manner [31]. In subjects living with MCI, authors showed that dysglycemia status is associated with lower brain glucose metabolism over time regardless of gender, associated, instead, with poorer executive function and language performance across time in women. These associations were not seen in men [31].

Considering the substantial evidence indicating gender-dependent variations in susceptibility to dementia and the gender-specific patterns observed in dementia prevalence, it becomes imperative to account for gender differences, particularly when evaluating the impact of dysglycemia on cognitive domains. Notably, epidemiological evidence shows that nearly two-thirds of individuals diagnosed with AD are women [32]. Some previous studies have also indicated that cognitive impairment is more prevalent among women diagnosed with T2DM compared to their male counterparts [10, 33]. In fact, women are particularly susceptible to diabetes-related declines in cognition with increasing age. However, an intriguing observation has also emerged, suggesting that women exhibit better cognitive performance irrespective of T2DM [34]. Considering that many studies did not include a reference group of people with dysglycemia, it remains unclear whether pre-diabetes is associated with greater presence of cognitive impairment and if it is more evident in women than in men. De Ritter and collaborators investigating cognitive performance in pre-diabetes showed no difference in any cognitive domains examined (verbal memory, processing speed, executive function, and attention) in either women or men [16]. Our findings, instead, suggest that the presence of dysglycemia differently impact on cognitive performances between genders.

Multiple linear regression analyses controlled for many confounding factors including age, years of education, vascular risk (evaluated by HIS), total cholesterol, LDL-cholesterol, HDL-cholesterol, triglycerides and antithrombotic drugs use revealed a significant association in the correlates of cognitive domains between genders. In detail in the presence of dysglycemia women, as compared with men, were more likely to show worse performances in Digit Span Forward test, a neuropsychological assessment tool that evaluates attention and short-term memory. This is in line with a previous finding showing that prediabetes worsens working memory in healthy older adults [35]. However, even in subjects without dysglycemia, among all neuropsychological tests, independent of multiple confounding factors, women showed poorer performances in Digit Span Forward as well as in ACER- Attention/Orientation and MMSE. These results further support the idea that older women performed worse than men in screening of general cognition as well as in some cognitive domains, including attention, orientation, and short-term memory. Thus, our findings suggest that there may be important between-gender differences in brain structure that might explain differences in cognitive functions.

Interestingly, despite the presence of dysglycemia women showed better performance in Rey Auditory Verbal Immediate and Delayed as well as in Categories Fluency Test as compared with men. The RAVLT assesses immediate and delayed memory for verbal material. It involves presenting a list of words and then evaluating how well the individual can recall them both immediately and after a delay. The Category Fluency Test, instead, is a neuropsychological assessment used to evaluate an individual’s executive function and verbal fluency. It specifically measures the ability to generate words within a specific category under timed conditions. It is possible to hypothesize that women in the later stages of mid-life exhibit superior cognitive performances in these domains compared to men as well as higher resilience to dysglycemia. Accordingly in a large cohort of adults with T2DM, there was a markedly lower prevalence of cognitive impairment (MCI or dementia) among women compared with men and correspondingly better performance in tests of cognitive function [36]. Endogenous estrogens are important for maintaining vascular function, repair of vascular damage, and promoting neurogenesis and may preserve cognitive functioning in older women [37, 38]. This study has several strengths, notably its large sample size and the utilization of comprehensive cognitive tests enabling the exploration of gender-specific effects on distinct cognitive domains. However, given the current cross-sectional analyses, it is important to understand whether these patterns continue over time or whether there are between-gender differences in cognitive trajectories.

In conclusion, while extensive research has explored the impact of T2DM on cognitive function, evidence regarding dysglycemia remains limited, particularly in relation to gender differences. Despite we confirm that the presence of dysglycemia is associated with a higher presence of neurocognitive abnormalities, including memory and logic reasoning, the natural history and clinical significance of these findings remain poorly defined. Notably, our study shows that women, especially with advancing age, exhibit a heightened susceptibility to deficits in overall cognitive function, attention, orientation, and short-term memory. The presence of dysglycemia further elevates the association of memory deficits in women. Although there were differences in the frequencies of common risk factors for cognitive impairment between genders, these did not account for differences in the association between women gender and cognitive performances. Moreover, despite the presence of dysglycemia women show higher ability in memory domains. This suggests that how dysglycemia may impact the progression of dementia, in the context of MCI, is related, at least in part, to female gender, independent of conventional risk factor relationships. The future research necessary to identify mechanisms that underlie this finding may lead to new targets for prevention and treatment, including the development of tailored interventions to reduce risks for cognitive impairment. Future research will be pivotal in elucidating the pathogenesis of cognitive dysfunction associated with prediabetes and its connection to gender. Although hyperglycemia and its resultant end-organ damage are implicated, the precise mechanisms through which hyperglycemia impacts cerebral structure and function remain unclear.

### Electronic Supplementary Material

Below is the link to the electronic supplementary material.

Supplementary Material 1

## Data Availability

No datasets were generated or analysed during the current study.
