# Supplementary material for: Dysglycemia, gender, and cognitive performance in older persons living with mild cognitive impairment: findings from a cross-sectional, population-based study
Source: Aging Clin Exp Res. 2024 Jul 16;36(1):145. doi: 10.1007/s40520-024-02806-7 (PMC11252216; doi:10.1007/s40520-024-02806-7)
Supplement: Supplementary file 1 — Supplementary Material 1 [file 40520_2024_2806_MOESM1_ESM.docx]

Supplemental Tables

**Supplemental Table 1.** Neuropsychological outcome measures

| DOMAIN | TEST | Lower score interpretation | Range scores | References Italian version |
| --- | --- | --- | --- | --- |
| *Attention* | Attentional Matrices | worse performance | 0-30 scores | Spinnler and Tognoni. 1987 |
|  | Trail Making Test A (TMT A) | better performance | 0-300 (time in seconds) | Amodio et al.. 2002[41] |
| *Memory* | Digit Span Forward | worse performance | No range | Monaco et al. 2013[42] |
|  | Digit Span Backward | worse performance | No range | Monaco et al. 2013[42] |
|  | Rey Auditory Verbal Learning Test Immediate and (RAVLT_I) Delayed recall (RAVLT_D) | worse performance | 0-75 scores | Carlesimo et al. 1996)[43] |
|  | Babcock story recall | worse performance | 0-16 scores | Spinnler and Tognoni. 1987[44] |
|  | Corsi Span | better performance | 0-10 scores | Spinnler and Tognoni. 1987 |
| *Language* | F-A-S Test (FAS) | worse performance | No range | Carlesimo et al. 1996) |
|  | Categories Fluency Test | worse performance | No range | Spinnler and Tognoni. 1987 |
|  | Token Test | worse performance | 0-36 scores | Spinnler and Tognoni. 1987 |
| *Executive Functions* | Trail Making Test B (TMT B) | better performance | 0-300 (time in seconds) | Amodio et al.. 2002[41] |
| *Fluid Intelligence*  *and logic reasoning* | Raven's Coloured Progressive Matrices (Raven’s Test) | worse performance | 0-36 scores | Carlesimo et al. 1996)[43] |

**Supplemental Table 2.** Clinical and biochemical characteristics of women population stratified by glycemic values (n=445).

|  | **Dysglycemia (n=121)** | **No dysglycemia (n=324)** | ***p*** |
| --- | --- | --- | --- |
| **Age (years)** | 78.50±7.38 | 74.72±10.12 | **<0.0001** |
| **Education (years)** | 8.69±4.73 | 9.91±5.16 | **0.025** |
| **DBP (mmHg)** | 76.13±10.52 | 73.54±10.34 | **0.033** |
| **SBP (mmHg)** | 135.02±17.54 | 129.79±16.76 | **0.008** |
| **Cholesterol total (mg/dl)** | 223.15±40.83 | 211.60±37.92 | **0.008** |
| **HDL-C (mg/dl)** | 61.73±13.41 | 63.50±15.41 | 0.291 |
| **LDL-C (mg/dl)** | 133.21±34.58 | 125.39±32.71 | 0.075 |
| **Triglycerides (mg/dl)** | 127.39±54.84 | 109.78±43.11 | **0.001** |
| **Glucose (mg/dl)** | 93.68±17.60 | 107.99±7.09 | **<0.0001** |
| **Vitamin B12 (pg/ml)** | 295.19±227.03 | 283.01±122.43 | 0.542 |
| **Albumin (g/dl)** | 4.00±0.00 | 4.07±0.26 | 0.494 |
| **Calcium (mg/dl)** | 9.80±0.42 | 9.57±0.64 | 0.339 |
| **Folic acid (ng/ml)** | 11.58±7.89 | 11.44±6.55 | 0.954 |
| **HIS** | 1.97±1.24 | 1.85±1.56 | 0.496 |

DBP: diastolic blood pressure; SBP: systolic blood pressure; HDL-C: high-density lipoprotein cholesterol; LDL-C: low-density lipoprotein cholesterol. HIS: Hachinski Ischemic Score.

**Supplemental Table 3.** Clinical and biochemical characteristics of men population stratified by glycemic values (n=237).

|  | **Dysglycemia (n=72)** | **No Dysglycemia**  **(n=165)** | ***p*** |
| --- | --- | --- | --- |
| **Age (years)** | 77.93±6.43 | 76.16±8.33 | 0.109 |
| **Education (years)** | 10.58±5.03 | 10.48±5.06 | 0.884 |
| **DBP (mmHg)** | 72.86±9.52 | 73.54±10.34 | 0.533 |
| **SBP (mmHg)** | 132.41±17.45 | 131.16±21.01 | 0.694 |
| **Cholesterol total (mg/dl)** | 188.96±35.64 | 192.95±39.2 | 0.477 |
| **HDL-C (mg/dl)** | 49.97±10.1 | 54.29±15.42 | 0.096 |
| **LDL-C (mg/dl)** | 118.06±32.71 | 116.5±35.05 | 0.786 |
| **Triglycerides (mg/dl)** | 110.79±47.87 | 109.47±50.39 | 0.857 |
| **Glucose (mg/dl)** | 108.28±7.33 | 97.27±19.99 | **<0.0001** |
| **Vitamin B12 (pg/ml)** | 245±124.68 | 300.99±263.37 | 0.111 |
| **Albumin (g/dl)** | 4.00±0.00 | 4.13±0.35 | 0.304 |
| **Calcium (mg/dl)** | 9.25±0.5 | 9.36±0.5 | 0.705 |
| **Folic acid (ng/ml)** | 11.43±7.8 | 7.4±6.09 | 0.250 |
| **HIS** | 2.59±1.44 | 2.08±2.05 | 0.082 |

DBP: diastolic blood pressure; SBP: systolic blood pressure; HDL-C: high-density lipoprotein cholesterol; LDL-C: low-density lipoprotein cholesterol. HIS: Hachinski Ischemic Score.
